# Patient Perspectives on Low-Dose Computed Tomography for Lung Cancer Screening, New Mexico, 2014

**DOI:** 10.5888/pcd13.160093

**Published:** 2016-08-18

**Authors:** Shiraz I. Mishra, Andrew L. Sussman, Ambroshia M. Murrietta, Christina M. Getrich, Robert Rhyne, Richard E. Crowell, Kathryn L. Taylor, Ellen J. Reifler, Pamela H. Wescott, Ali I. Saeed, Richard M. Hoffman

**Affiliations:** Author Affiliations: Andrew L. Sussman, Robert Rhyne, Department of Family and Community Medicine, University of New Mexico School of Medicine, Albuquerque, New Mexico; Ambroshia M. Murrietta, Clinical and Translational Science Center, University of New Mexico, Albuquerque, New Mexico; Christina M. Getrich, Department of Anthropology, University of Maryland, College Park, Maryland; Richard E. Crowell, University of New Mexico Comprehensive Cancer Center, Department of Internal Medicine, University of New Mexico School of Medicine, Albuquerque, New Mexico; Kathryn L. Taylor, Department of Oncology, Cancer Prevention and Control Program, Lombardi Comprehensive Cancer Center, Georgetown University Medical Center, Washington, DC; Ellen J. Reifler, Pamela H. Wescott, Informed Medical Decisions Foundation/Healthwise, Boston, Massachusetts; Ali I. Saeed, Division of Pulmonary Critical Care and Sleep Medicine, Mayo Clinic, Rochester, Minnesota; Richard M. Hoffman, Department of Medicine, University of Iowa Carver College of Medicine, University of Iowa Holden Comprehensive Cancer Center, Iowa City, IA.

## Abstract

**Introduction:**

National guidelines call for annual lung cancer screening for high-risk smokers using low-dose computed tomography (LDCT). The objective of our study was to characterize patient knowledge and attitudes about lung cancer screening, smoking cessation, and shared decision making by patient and health care provider.

**Methods:**

We conducted semistructured qualitative interviews with patients with histories of heavy smoking who received care at a Federally Qualified Health Center (FQHC Clinic) and at a comprehensive cancer center-affiliated chest clinic (Chest Clinic) in Albuquerque, New Mexico. The interviews, conducted from February through September 2014, focused on perceptions about health screening, knowledge and attitudes about LDCT screening, and preferences regarding decision aids. We used a systematic iterative analytic process to identify preliminary and emergent themes and to create a coding structure.

**Results:**

We reached thematic saturation after 22 interviews (10 at the FQHC Clinic, 12 at the Chest Clinic). Most patients were unaware of LDCT screening for lung cancer but were receptive to the test. Some smokers said they would consider quitting smoking if their screening result were positive. Concerns regarding screening were cost, radiation exposure, and transportation issues. To support decision making, most patients said they preferred one-on-one discussions with a provider. They also valued decision support tools (print materials, videos), but raised concerns about readability and Internet access.

**Conclusion:**

Implementing lung cancer screening in sociodemographically diverse populations poses significant challenges. The value of tobacco cessation counseling cannot be overemphasized. Effective interventions for shared decision making to undergo lung cancer screening will need the active engagement of health care providers and will require the use of accessible decision aids designed for people with low health literacy.

## Introduction

Despite advances in treatment and management, lung cancer remains the most common cause of cancer-related disease and death in the United States (1). Most lung cancer deaths are attributed to smoking (2). Although recently declining smoking rates may reduce lung cancer incidence (1), former smokers remain at a high risk for the disease, and most lung cancers occur in former smokers (3).

Until recently, no effective screening method existed for detecting early-stage lung cancer (4). The multicenter National Lung Screening Trial (NLST) enrolled 53,454 high-risk smokers, defined as people aged 55 to 74 years with a 30 pack year smoking history, who were either current smokers or had quit within the previous 15 years (5). Lung cancer deaths among NLST participants randomly assigned to low-dose computed tomography (LDCT) were significantly reduced (16.0% [relative] (6) and 0.33% [absolute] (5) risk decrease) compared with those who had chest x-ray. However, concerns remain regarding the high false positive rates and complications following invasive diagnostic procedures (ie, percutaneous biopsy, bronchoscopy, or surgical procedure) (5).

Based on the NLST results, the United States Preventive Services Task Force (USPSTF) issued a Grade B recommendation supporting LDCT screening for high-risk adults from 55 to 80 years of age (7). The recommendation is important since the Affordable Care Act mandates coverage without copayment for preventive services that have a USPSTF recommendation of B or higher (8). Guided by the USPSTF recommendation, the Centers for Medicare and Medicaid Services (CMS) now provide annual coverage for lung cancer screening, with a requirement of a shared decision-making visit (ie, consultation between patient and health care provider), for beneficiaries meeting NLST eligibility criteria (9). The CMS directive also stipulates smoking cessation counseling and the use of decision aid(s) to discuss potential screening outcomes (9). The American Lung Association (10) and the American Cancer Society (11) also endorsed lung cancer screening for patients meeting the NLST eligibility criteria. Both organizations recommended both informed and shared decision making with clinicians and smoking cessation counseling (10,11).

Although there is a need to promote shared decision making for lung cancer screening (10,11), gaps exist in our understanding about how best to concisely and clearly present complex probability-based screening information (eg, lung cancer risk, false positives, consequences of diagnostic follow-up), while effectively integrating smoking cessation messages. Moreover, it is important to better understand the perspective of persons who are demographically different from those included in the NLST, the majority of whom were non-Hispanic white (91%) and male (59%) (5). To develop appropriate decision-support strategies, perspectives of people from diverse sociodemographic backgrounds need to be incorporated. New Mexico, as one of the 4 “majority–minority” states, features unique multiethnic and multicultural diversity with striking health and socioeconomic disparities. Lung cancer is a leading cause of cancer death among minority populations in New Mexico (12). We interviewed patients in clinics to characterize their knowledge and attitudes about LDCT lung cancer screening and smoking cessation and their views on supporting decision making for lung cancer screening. We previously reported on primary care providers’ perspectives about implementing LDCT screening (13).

## Methods

### Study setting and sample

We conducted qualitative semistructured interviews with a diverse sample of patients who were receiving care at one of two settings in Albuquerque, New Mexico: a Federally Qualified Health Center (FQHC Clinic) or the University of New Mexico Comprehensive Cancer Center (UNMCCC) Multidisciplinary Chest Clinic (Chest Clinic). In New Mexico, the University of New Mexico in Albuquerque is the only academic medical center and is host to the only National Cancer Institute Comprehensive Cancer Center in the state. This poses access issues for rural patients. At the time of this study, neither the FQHC Clinic nor the Chest Clinic offered LDCT for lung cancer screening, nor were we aware of any comprehensive screening program in the state. The FQHC Clinic is part of the Research Involving Outpatient Settings Network (RIOS Net), the Practice-Based Research Network of New Mexico that predominantly serves Hispanic patients. By using a purposeful sampling approach, we identified, recruited, and interviewed adults aged 50 to 80 years with a history of heavy smoking who met NLST enrollment criteria (5). Eligible participants recruited from the FQHC Clinic self-reported not having lung cancer or nodules and not having undergone a lung computed tomography (CT) scan. Those recruited from the Chest Clinic were documented to have had abnormal results from a lung CT scan. Selecting a wide spectrum of at-risk patients enabled us to include perspectives of patients ranging from those considering screening to those who actually underwent the imaging and invasive diagnostic testing that would be part of a screening program. These perspectives were necessary to develop comprehensive decision support tools to inform patients about screening and surveillance testing and associated invasive diagnostic procedures. The University of New Mexico Health Science Center’s Human Research Protections Office approved all aspects of the research protocol.

### Data collection

We developed an interview guide that focused on the domains of knowledge and attitudes regarding LDCT lung cancer screening: perceived risk from diagnostic testing (including radiation exposure and complications from invasive diagnostic tests), views about the benefits and harms of lung cancer screening, and views about barriers and facilitators to smoking cessation or preventing relapse, particularly in the context of lung cancer screening. We also elicited views about the value, content, format, and timing of providing patient decision-making aids and preferences for discussing screening. Before the interview, we administered a survey that collected sociodemographic information. The survey and interview guide are available from the corresponding author.

Three members of the research team (A.L.S., A.M.M., C.M.G.) conducted the interviews from February through September 2014. Each digitally audio-recorded interview lasted from 45 to 60 minutes and was transcribed for analytic review. We conducted in-person interviews at the participant’s choice of location. Each participant received a $50 merchandise card.

### Data analysis

We used a systematic iterative process to identify preliminary and emergent themes of importance (14). Several research team members independently read sets of 2 or 3 transcripts to identify preliminary findings (S.I.M., A.L.S., A.M.M., C.M.G., R.R., R.M.H.), which facilitated confirmation of emergent themes and modifications to the interview guide. Through an iterative process of reviewing additional transcripts, we created an initial coding structure that was then revised until we reached consensus (15). We then imported all the transcripts into NVivo 10 (QSR International), a qualitative data analysis software program, for coding. After coding all transcripts, we queried the database by coding categories for a more refined level of interpretive analysis. We analyzed the quantitative survey data to produce a descriptive assessment of the study sample.

## Results

### Sociodemographic characteristics of participants and their views on cancer screening

We interviewed 22 clinic patients, 12 from Chest Clinic and 10 from FQHC Clinic. Table 1 presents their sociodemographic characteristics. We categorized the qualitative interview findings into major themes, which were described by FQHC and Chest Clinic participants (Table 2). Participants in both clinics endorsed the general importance of screening for health problems, even if they personally did not undergo regular screening, because it “may reveal something unpleasant, but still it is good to know” to “catch things early.” Some participants previously underwent cancer screening tests such as colonoscopy, mammography, or Pap smear; for one participant, this led to early cancer diagnosis and treatment. Not surprisingly, participants identified barriers to engaging in screening such as the cost of purchasing insurance, lack of transportation, and low health literacy (eg, “they use big words and I don’t know what they mean”). They also generally noted that screening was not a normal behavior among their family and social networks.

**Table 1:** Participant (N = 22) Characteristics, Study of Patient Perspectives on Low-Dose Computed Tomography for Lung Cancer Screening, New Mexico, 2014

Characteristic	Chest Clinic (n = 12), n	FQHC Clinic (n = 10), n	Clinics Combined (n = 22),
**Age, mean (SD)**	61.3 (10.31)	55.5 (3.98)	58.6 (8.43)
**Sex**			
Male	6	7	13
Female	6	3	9
**Race/ethnicity**			
Hispanic	5	9	14
White	6	1	7
African American	1	—	1
**Marital status**			
Married or living with partner	5	1	6
Separated, divorced, widowed, never married	7	9	16
**Employment**			
Employed (full- or part-time)	5	1	6
Unemployed	4	7	11
Retired	3	1	4
Refused to answer	—	1	1
**Education**			
12 years or less	6	9	15
More than 12 years	6	1	7
**Income**			
Less than $20,000	5	9	14
$20,000 or more	7	1	8
**Insurance status**			
Medicaid	6	10	16
Medicare	4	—	4
Private	1	—	1
None	1	—	1
**Currently use tobacco products**			
Yes	3	6	9
No	9	4	13

Abbreviations: —, not available; chest clinic, a comprehensive cancer center-affiliated chest clinic; FQHC, Federally Qualified Health Center; SD, standard deviation.

**Table 2:** Examples of Participant Comments About Lung Cancer Screening, by Clinic Type, Study of Patients’ Perspectives on Low-Dose Computed Tomography for Lung Cancer Screening, New Mexico, 2014

Theme	Chest Clinic	FQHC Clinic
**General views on preventive care (screening)**	•“[It’s] especially like when you’re maybe 35, 40 years old, that’s when people start opening their eyes. I think that as you get older, people are more interested in, ‘yeah, let’s go check something out.’” “Well, I think that those are great. I get a colonoscopy every five years and a mammogram every year.” “I think more people would probably participate providing they can afford it. When you mention medical to anybody, the first thing they think of is, ‘here goes the money.’” “Like my husband, he’s ‘oh, I’m superman. I don’t need to go to the doctor’s’ and I’m telling him, ‘you don’t know that, you may think everything’s fine.’”	“I’ve done mammograms, and I think that’s a good thing for the women to do. I tell my daughter the same thing. I go, ‘you need to take care of yourself.’” “I had a . . . what do you call it? Borderline cancer of my cervix at one time. I was younger, and I went for my Pap and they told me I had an abnormal Pap.” “I really, actually I don’t like doctors. I get scared about what they’re gonna tell me, but now I’m at this age where I’m having these symptoms and these things that are happening to me. I’m more concerned about myself so I need to get help.” “No, they [family members] don’t go to the doctor as much as I do.”
**Knowledge and receptivity to LCDT screening for lung cancer**	“I’m just now learning that they use chest x-ray and CT. Thirty years ago, I’d say, ‘Ah, bullshit.’ I would usually tell somebody, thanks, but I’m not interested. But I thought about it and you know what? I’ll be generous enough to at least give you that.” “[prior to a diagnosis of breast cancer, she would have said] ‘I don’t need it.’ You know? I mean, that’s just it, you don’t really think about it. You know? Until something happens.” “I don’t think so. Oh, absolutely. Especially at my age. I think it alleviates uncertainty. And when you’re referred and scheduled for one of these, you really need to have answers. I don’t work well with uncertainties.” “There’s got to be some type of a screening for us smokers, because when I went in last year, I didn’t have the spot on my lung and if it was screened, it wouldn’t have gotten as big as it did and I didn’t have symptoms and I still don’t. But I have lung cancer. So CT screening would help, especially if you’re a smoker.”	“Yes, I would if I can afford it. I’d rather do it because I want to know what’s wrong with my health so we can fix it.” “No [have not heard about it]. This is what I need. Bad. You know it’s one thing to have a breathing problem, but it’s one thing to know that without a referral you could get a scan and check your lungs. Uh, accuracy. Benefit of the doubt. You know straight up what’s wrong, what’s there and what’s not.” “Uh . . . for lung cancer, no . . . I can’t believe I [have heard about the test]. I’d have to think about it. I don’t know. I’d have to weigh it out. I’d have to do the do’s and don’ts and I don’t know. I have to think about it and who knows, I probably would.” “No [not heard]. No, I don’t think I want to do it. To be doing it every year and every year, it’s gonna stay on your head. Every year you can say you know, what’s gonna happen, what’s gonna happen?”
**Challenges to LDCT screening for lung cancer**	“Well I would think about the risks, depending on what it was, but I have a lot of faith in my providers, I think I have really good providers.” “The false readings. The continued tests that aren’t necessary.” “The cost ‘cause nowadays, I mean, everything’s so expensive. And if I have insurance, the insurance is gonna cover it, it’ll be easier because, I mean, the CT scan is what, about $1000? You have to have insurance, right? It’s money. And if we don’t have the money to pay for the treatment or what the case may be, you make it hard.” “Like here in New Mexico that you are living in the middle of nowhere. You have to travel 150 miles to get to the hospital. I can see that those kind of people, especially since the income rate within the state is as low as it is, especially for older people which would be the type of people that need this, and the . . . equipment, and technology that you need in order to do the low dose scans isn’t something that you can just roll out there in the middle of nowhere.”	“If I can afford it. I wouldn’t be able to get it if I had to pay for it. I’d have some concern if I had to buy certain pills for it out of my pocket; I wouldn’t be able to afford it.” “False positives. Long screening, yeah, that’s a long time. The radiation, too ‘cause I heard a lot of bad things about radiation. The stress and the anxiety. Yeah, ‘cause I would be thinking about that all the time it’s going on and I don’t even like to go to doctor.” “That I know I won’t get no cancer from the radiation. That’s my main concern. You already explained to me that it’s light, it’s not a heavy radiation, so that’s appealing to me in a positive way as far as me doing it but, if it was like a high risk, you know, I’d be like, no. No, they [false positives] don’t bother me because I think that there’s a 50/50 chance.” “What my health is that’d be my most important. If I’m sick I want to know. The risk of how much that [radiation] would jeopardize my health, too. I mean how much radiation are they gonna give me? What is it gonna do to me, the side effects, you know? That [annual screening] wouldn’t be a problem.”
**Smoking cessation in the context of LDCT for lung cancer**	“No, and I think most people would be in that same boat. You know, they got good news [ie, lungs are fine], so it didn’t help them change nothing [ie, quit smoking]” “No, I don’t think so. I think at that point I probably would have still lit up the day before I went or the day of going to the test. I would have probably thought about it after I had gotten the CT scan and seeing what the results were. That’s just where your mind’s set at and nothing scares you until you hear that word, that big C word.” “I would most likely keep smoking until they tell me that you have to stop or this is what’s gonna happen. And if I go once a year and if it shows I’m okay, most likely I’ll be smoking. If they tell me it’s cancerous, now it’s like a rude awakening. You have to stop.” “Exactly and that’s how I would feel. That oh, that’s great. Being honest with myself, if they told me today, ‘You don’t have lung cancer,’ I wouldn’t go back and pick up the cigarettes. I went through hell to get rid of them. But people that are smoking were told, ‘No, your lungs are just fine. There’s nothing wrong with them.’ I think most people would go, ‘Whew. I thought they were going to tell me I had to quit.’”	“I don’t know. I think when somebody wants to quit they’ve gotta just want to quit. I probably would [quit] if you showed me how bad my lungs were and what I’ll look like in 3 more years.” “Oh hell yeah, that would tell me yay or nay, throw it away. [If negative result] That means that I’m really not a problem smoker. [If positive result] Just smoke a cigarette and think about what it is, at least it’s got my attention. You know like let’s say if it wasn’t quitting smoking and it’s only getting worse, was the smoking keeping it at a minimum? I would probably sit there and give it the 6-month lap. That means I would still smoke, but a lot, lot less, so by the time the last 2 months of that 6 months come, I would be not smoking.” “[If positive result] I hope it would really sink inside me and say, ‘hey, you know what? You’ve got to quit’. [If negative result] No, I think I would want to still quit. Because I say that, ‘hey, next week, boom, I could just get it.’” “Yes. I’ll get a better understanding and a better reading about what’s on the CT scan. I’ll get more feedback from a doctor, from a professional. I think it would benefit me and help me stop smoking even more if I knew that, that there is complications that are going on.”
**Information needs and preferred communication methods**	“One-on-one, is the way that I want my information. I want my information to come directly from my doctor or from the nurse that I’m working with or the clinic I’m working with.” “Probably the percentages of . . . the false readings. The results of what happens when they get false readings. Probably printed material. Face time with doctor.” “All the information I could get . . . and in plain words, which I could probably understand it, that would be the best. Information about the test and the consequences and the procedure itself time-wise. Most people do sit down a lot and watch movies or DVDs. I think they would just like a 15 minute explanation of the procedure, how it’s done, why this is being done, what kind of machine is gonna be used. The doctor is the actual one that’s gonna give you the results.” “I think there should be a lot more, instead of the smoking cessation like the 1-800-QUIT NOW. They showed the guy with the hole in the throat, the woman with the lung removed, things like that, and then after that could be an informative little message about if you [smoke] . . . this could be prevented with a CT scan now. [A] family member should be involved or family member to come with them, ‘cause the family member will most likely talk them into it. For me I want to know from the doctor. I think pamphlets in the doctor’s office on the wall, like they have other pamphlets there, because the longer you wait, the more you get bored and the more you read.”	“Just show me pictures of lungs. People on more medication. When I come into my appointments and I’m sitting down, that’s how I read. Sometimes people won’t come to doctors, because they just have a thing; for them I would say grocery stores, where the ads are for the *Quick Quarter *or the cars. Pamphlet there or something for smokers.” “All the risk and the cost, but mostly the risk. “Maybe on a DVD or something so you could watch, ‘cause sometimes a lot of people don’t like to read. Like I don’t like to really read. The doctor, too, but sometimes the doctors have certain amount of time to talk to you. Sometimes some people don’t have [time] for the Internet. They don’t have computers, I don’t have a computer.” “The correct information, the information that is stated on a piece of paper and the information that is stated from a professional. Information about the procedure, risks, benefits, future treatment, side effects. A video, you know like a video going through a facility like we’re here and you pull out a video and say, ‘look, this is what it consists of.’ It could be a trained health professional.” “I know I’ve smoked for a long time. I don’t really know what my lungs look like. If the people knew how bad their lungs were, maybe they would think about it a little more. Pretty much straight from my doctor, yeah I’d rather hear from him. Reading it and stuff really just doesn’t hit you as like the doctor telling you. You might have a question that you want to ask, and with a video you won’t be able to ask any questions.”

Abbreviations: Chest Clinic: a comprehensive cancer center-affiliated chest clinic; CT, computed tomography; DVD, digital versatile disk; FQHC, Federally Qualified Health Center; LDCT, low-dose computed tomography.

### Knowledge and receptivity to LDCT screening for lung cancer

None of the participants interviewed at the FQHC Clinic had heard about using LDCT to screen for lung cancer. During the interview, we provided a brief explanation of LDCT screening, and most participants were interested. Participants who were either uncertain or conditional about whether they would undergo lung cancer screening expressed uncertainty about the value of screening (in terms of the benefits and necessity) and logistics of the screening procedure. Other participants were favorably inclined toward screening but were still concerned about costs and the accuracy of the test. Those strongly indicating that they would undergo screening were concerned for their health given the known consequences of long-term tobacco use.

Most Chest Clinic participants were unaware of LDCT screening. However, receptivity to receiving the test was generally high, especially if the test was recommended by a health care provider. Several participants reported being more inclined to undergo LDCT screening (and other screening tests) because of a general sense of vulnerability that they attributed to their current situation (ie, being under surveillance for lung cancer or having a diagnosis of lung cancer) or their experience with other health problems.

### Challenges to LDCT screening for lung cancer

In discussions with FQHC Clinic participants concerns about high costs, radiation exposure, and psychological distress (stress, anxiety) all emerged as considerations about whether to undergo LDCT screening. Most participants were not dissuaded by the potential for false positives and the need for continuous followup and screening. We provided a brief explanation of LDCT screening but did not review specific details (eg, false positives, followup testing for positive scans). However, based on interviewer debriefing notes following the discussions, it was unclear whether all participants fully understood the meaning and implications of these terms (eg, false positives, followup testing for positive scans).

Chest Clinic participants identified several factors that could pose challenges to LDCT screening. They were particularly mindful about costs but said they would make sacrifices to obtain necessary care. As one participant noted, “I need to know if I have it or not . . . I’ll deal with the money part of issues and aftercare when I get to that.” Other challenges mentioned were the cost of treatment, transportation issues, distance to the screening site, being able to trust health care providers, taking tests that were inconclusive or invasive, and the need for annual screening. Participants were generally not concerned about radiation exposure, false positives, or the need for continued follow-up and screening.

### Smoking cessation in the context of LDCT for lung cancer

We also explored views about how LDCT results — either positive or negative — might influence smoking behavior. FQHC Clinic participants indicated that undergoing screening by itself might not be a strong deterrent to smoking. However, several felt that a positive test result might be sufficient motivation for a quit attempt. Participants further noted that actually seeing a lung nodule on an image would serve as a more powerful message that something is wrong than just being told of a problem. Several indicated that even a normal test result would not be sufficiently reassuring and that they would still consider quitting smoking. However, participants expressing a fatalistic worldview might not be swayed into considering quitting. Chest Clinic participants expressed similar views about LDCT screening and smoking cessation. Of these 12 participants, 9 were no longer smokers and 5 had quit smoking because of a major scare (eg, receiving test results about a suspicious lung nodule, having a friend or family member with diagnosed lung cancer). Three Chest Clinic participants had quit smoking since learning about a positive finding on an x-ray or CT scan leading them to be seen at the Chest Clinic. However, several participants believed that a negative screening result might not be sufficient to persuade someone to quit smoking. Conversely, a few participants lamented that such a finding could have the opposite effect and serve as a green light to continue smoking.

### Information needs and preferred communication methods

After describing LDCT screening to participants, we then asked them for input about the most important information to include in a decision-making aid. FQHC Clinic participants suggested pictures of a lung damaged by smoking, general information about lung cancer, benefits and harms of LDCT screening, cost and duration of screening, and potential future treatment and treatment side effects. Participants preferred learning about the screening test in a face-to-face encounter with their health care provider. Participants also suggested pamphlets or written materials; however, they raised concerns about poor literacy and inability to understand information. Others suggested using DVDs or CDs, which someone could view in a doctor’s office and then seek clarification from the doctor. They felt that a Web-based decision aid might not be effective because of limited access to computers and the Internet, a concern with particular relevance in a low-income and rural state such as New Mexico.

Chest Clinic participants provided greater specificity regarding the types of information that they would like to see in a decision aid, including information about LDCT, risks and benefits of screening, false positives and negatives, and consequences of undergoing the test. These participants preferred to receive screening information through one-on-one discussions with a health care provider. Other suggested information sources included booklets (with pictures), videos, DVDs, and social media.

## Discussion 

This study presents findings from in-person qualitative interviews of high-risk patients, with or without lung cancer, regarding LDCT screening for lung cancer, smoking cessation, and decision-making support. The views summarized in this article are some of the first from participants who were underrepresented in the NLST and from patients not considered eligible by the NLST criteria (ie, patients with a lung nodule or lung cancer). These interviews provided insights into the screening process and perceptions regarding smoking cessation within the context of screening. The findings offer insights into the relative value of focusing on smoking cessation counseling in the context of lung cancer screening, challenges to offering LDCT screening to sociodemographically diverse populations, and the content and structure of decision aids.

A consistent message from guidelines (9–11) is that patients be offered smoking cessation counseling along with lung cancer screening. Our findings provide evidence in support of these guidelines. Our findings suggest that smokers might attach personal benefits to lung cancer screening results, with negative findings implying “got good news” and “I’m not a problem smoker” and positive findings potentially motivating smokers to quit (16). Zeliadt et al (17), based on a qualitative study conducted at Veterans Health Administration sites, also reported that negative screening findings lowered participants’ motivation for cessation. Meanwhile, the Chest Clinic patients who had quit smoking all cited the powerful effect of an abnormal finding, and many of the FQHC Clinic smokers thought that abnormal findings could motivate cessation. Indeed, the NLST reported that CT findings highly suspicious for cancer were strongly associated with subsequent smoking cessation (18). Our findings point to the importance of providing referrals for evidence-based smoking cessation interventions (16) and also integrating smoking cessation counseling when offering screening, discussing the results, and using these doctor-patient interactions as a teachable moment for cessation counseling (19); primary care providers in a parallel arm of this study endorsed this referral and counseling approach (13). Ultimately, achieving smoking cessation is likely to have a far greater public health benefit than screening alone.

Another recommendation from the lung cancer screening guidelines (9–11) is the use of decision aids and shared decision making between patient and physician. The lack of awareness about lung cancer screening coupled with high receptivity for the test among high-risk smokers underscores the need for decision aids. Before our study, however, there was no evidence to guide the content and structure of decision aids for lung cancer screening tailored for populations not recruited in the NLST. Our findings provide evidence for inclusion of key areas in decision aids, including a basic description of the LDCT screening test and how it is conducted, benefits and harms of screening, sequence of events following an abnormal screen, visual depiction of damage to the lungs from smoking, lungs with nodules or cancer, estimated cost of screening and follow-up procedures, and a clear statement about the benefits of smoking cessation regardless of screening results. Finally, our findings provide evidence for the need for shared decision making and the need for health care providers to actively engage eligible patients about smoking cessation and lung cancer screening through one-on-one interactions. The shared decision-making discussions should be supplemented with written and audiovisual (CD or DVD) aids to circumvent reading and health literacy constraints, and these discussions should be tailored to individual patient concerns and circumstances. A patient decision aid (20) is being evaluated in a Patient-Centered Outcomes Research Institute grant (21), and a Web-based (22) patient decision aid needs further study to determine its efficacy and generalizability.

Offering LDCT screening for lung cancer poses immense challenges. It is well-documented that participation in cancer screening is lower among racial/ethnic minorities than whites (23) and is positively associated with cancer-specific and screening-specific knowledge (24), proximity to screening centers (23), years of education (23), high income (23), residence in urban areas (25), social support and normative behaviors that promote preventive behaviors (26), and private insurance (23). These positive predictors for screening do not typify participants in our study who were predominantly Hispanic, unemployed, with low levels of education, less normative behavior for preventive care, and on government insurance (Medicaid). Furthermore, participants were unaware of the existence of the LDCT screening test and identified additional barriers to screening such as high costs, transportation barriers, need for annual screening, and psychological distress. Therefore, despite participants’ indicating their receptivity to LDCT screening, sociodemographic and psychosocial barriers may preclude equitable delivery and uptake of lung cancer screening and may further perpetuate lung cancer disparities.

The health equity issues related to people who do not resemble those enrolled in the NLST may also extend to geographic areas. Guidelines are clear that screening should not be offered except through centers of excellence (7,11). Most NLST sites were university or academic cancer centers. Other hospital centers in New Mexico may not have sufficient resources and expertise to meet the criteria necessary to offer lung cancer screening. This could result in patchy national availability of lung cancer screening that could further exacerbate disparities. The number of institutions nationally having an active LDCT screening program are increasing (27); however, concerns exist regarding screening use, infrastructure and resources to handle screening demands, and compliance with recommended guidelines. Possible solutions could be to rely more on telemedicine to ensure high-quality image interpretations and to develop regional centers of excellence that could serve a broader population. Applying more stringent criteria for screening could also be beneficial by increasing the efficiency of screening. A post-hoc analysis of the NLST data showed that the 3 highest quintiles of risk accounted for 88% of the screening-prevented lung-cancer deaths (28).

Our study has strengths and limitations. The findings from this qualitative study are not representative or generalizable to other types of clinical settings and do not represent the unique belief systems and needs of sociodemographically diverse populations. The results reported here are consistent and contextually relevant perceptions of smoking cessation and lung cancer screening from 2 groups of high-risk patients and are necessary to guide future efforts toward implementing LDCT screening, especially in populations and communities unlike those featured in the NLST study. Although the National Cancer Institute excluded people with a history of lung cancer from the NLST, the American Association for Thoracic Surgery (29) and the National Comprehensive Cancer Network (30) recommend screening for lung cancer survivors. Participants in our study represent a challenging population to screen, especially in terms of access to care, health literacy, and insurance coverage. These participants and associated findings further underscore challenges in translating research into practice.

Implementing lung cancer screening programs among sociodemographically diverse populations will be challenging and could further perpetuate lung cancer disparities. Value of smoking cessation counseling for current smokers within the context of lung cancer screening cannot be overemphasized, especially in light of the evidence that positive screening findings might be viewed as a motivation to quit. Screening programs will need literacy-appropriate decision aids coupled with smoking cessation counseling offered independently or conjointly in the context of lung cancer screening. Policy makers should consider allocating greater resources to smoking cessation interventions, especially in locations where LDCT screening services cannot be offered in compliance with recommended guidelines (9–11).
